# Compact Optical Visual Magnification System with a Wide Field of View

**DOI:** 10.3390/s25227025

**Published:** 2025-11-17

**Authors:** Uri Milman, Jacob Kaufmann, Yoel Arieli

**Affiliations:** The Jerusalem College of Technology, Jerusalem P.O. Box 3062, Israel; uri.mil17@gmail.com (U.M.); implant@012.net.il (J.K.)

**Keywords:** optical design, visual magnification, field of view, power corrector, telescope, binoculars, eyepiece design, aberration correction, compact optics, wide-angle optics

## Abstract

**Highlights:**

**What are the main findings?**

- A Power-Corrector (PC) group positioned between the objective and intermediate image enables unified optimization of the optical system, achieving superior performance with an 86° apparent field of view while maintaining compact dimensions.
- The PC-based design demonstrates significantly improved optical quality with ~5% distortion compared to ~24% in conventional designs and achieves 42.5% length reduction and 44.9% diameter reduction compared to Nagler-based systems with identical specifications.

**What is the implication of the main finding?**

- The PC group enables practical wide-field optical devices with 1.6× larger FOV diameter (~2.56× viewing area) than conventional monoculars of identical physical dimensions, enhancing scene scanning efficiency for applications requiring stationary observation with eye movement scanning.
- This technology enables compact, high-performance monoculars, binoculars, and spotting scopes suitable for bird watching, hunting, sports, military, and astronomical applications, addressing the traditional trade-off between field of view and system compactness.

**Abstract:**

A new concept for designing a visual magnification system is introduced. In this concept, apart from the conventional objective and the eyepiece group lenses in the system, an additional Power-Corrector (PC) group of lenses is introduced in the optical design. The Power-Corrector group is located between the objective and the intermediate image and allows an additional degree of freedom to the optical design. This enables improving the system’s performance such as the field of view (FOV) while eliminating the system’s aberrations or reducing them to an insignificant level. Incorporating the PC group achieves this by correcting aberrations that typically limit the performance of conventional eyepiece designs, allowing a wider acceptance angle for incoming light. Recent advances in wide field of view optical systems and compact optical design methodologies have highlighted the ongoing challenges in balancing field of view expansion with aberration control. In this configuration, the PC group unifies the units, the eyepiece and the objective groups, integrates their functionality to one coherent system such that the input focal length of the system is defined jointly by the focal lengths of the objective and the PC while the aberrations are corrected jointly by the PC and the eyepiece. Thus, while the system’s magnification is the ratio of the input focal length to the eyepiece focal length, the PC enables global optimization such that the PC and the eyepiece together have a combined aberration that is less than the characteristic aberration of the eyepiece. This integrated optimization enhances the FOV. Additionally, it maintains image quality, making the system more effective than traditional designs. Contemporary research in freeform optical surface design and optimization techniques demonstrates the growing importance of advanced aberration correction methods in modern optical systems. Using this concept, a compact imaging system with a wider FOV relative to customary designs with the same magnifications, was designed and manufactured.

## 1. Introduction

The Keplerian telescope, commonly used for magnifying visual images, consists of an objective group lens and an eyepiece group. To erect the inverted image, an inversion unit is typically utilized. When designing a visual observation optical system, key parameters such as the field of view (FOV) and magnification must be carefully considered. A large FOV is crucial for efficient scene scanning, detailed visualization, and concurrent observation of dynamic events. Conversely, a restricted FOV can pose challenges, especially in binoculars. Balancing FOV and magnification involves a tradeoff, with some systems maintaining a constant product of these two factors. Consequently, enhancing the FOV while preserving the magnification can result in a larger and heavier system. Moreover, factors like Exit Pupil (ExP) diameter and Eye Relief (ER) are also essential considerations [1,2].

In general, the exit pupil diameter is about 3.5–5 mm for telescopes that are used during daylight. The ExP diameter is derived from the entrance pupil diameter and the magnification. Maintaining adequate ER is also crucial for eyeglass wearers, as their eyes are typically positioned farther from the eyepiece [3].

**Figure 1:**
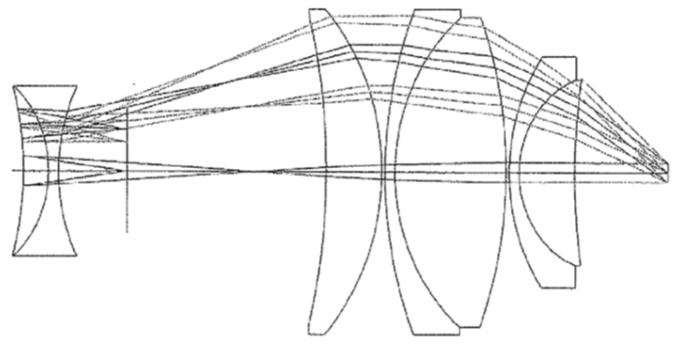
The Nagler eyepiece.

**Figure 2:**
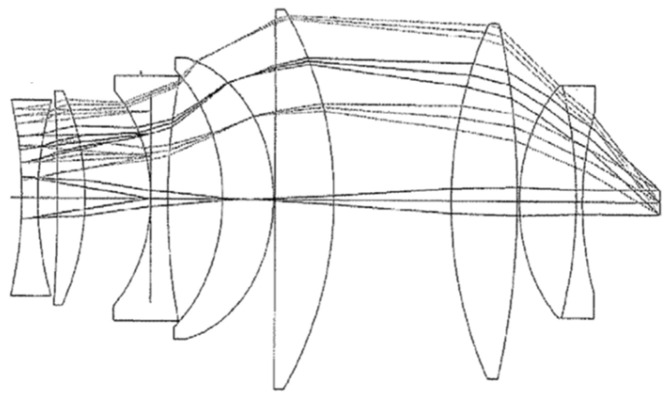
The Dilworth eyepiece.

A typical eyepiece with large FOV consists of two groups of lenses where one has negative power and the other has positive power. The major role of the negative power group, which is located before the intermediate image from the objective side, is to correct the distortion and the astigmatism while the positive power group is the main part of the eyepiece and is responsible for the positive power of the eyepiece. Both groups correct together the field curvature and increase the ER. In recent years, there have been developments of various types of eyepieces with large FOV [4]. One of the earliest and most popular large FOV eyepiece designs was the Nagler [5], which is illustrated in Figure 1. Additionally, the Dilworth eyepiece, shown in Figure 2, is another widely used and important large FOV eyepiece design that has been a significant development in this area [6].

In both designs, to achieve a large FOV, the rays enter the positive group lenses of the eyepieces at significant ascending angles and exit with sizable descending angles. As a result of these angles, the eyepiece has a large diameter, approximately 60–100 mm. The negative element positioned in front of the image plane in both designs is commonly referred to as the Smyth lens. The negative element aids in reducing the Petzval sum of the eyepiece, functioning as a field-flattener lens and enhancing ER. However, some of the disadvantages of these designs are that they are not compact, are heavy, and require many different lens elements. Recent advances in wide field of view optical systems [7] and in compact optical system design [8,9] have explored diverse technological pathways at multiple levels. At the material and surface level, advanced non-rotationally symmetric surface geometries [10,11,12] and exotic freeform gradient-index media [13] offer powerful aberration correction capabilities, with applications demonstrated in compact off-axis systems [8], desensitization methods [14], spectral imaging [15], miniaturized high-resolution systems [16], and error-tolerant designs [17]. Computational optimization via differentiable ray tracing [18] has further expanded design possibilities. At the component level, meta-optics solutions for AR/VR applications [7,19] represent alternative approaches. The Power Corrector (PC) group architecture presented here demonstrates that significant size reduction and wide-field performance can be achieved through system-level innovation using conventional spherical optics throughout, offering a practical, immediately manufacturable pathway to compact wide-field visual observation systems.

This article introduces an advantageous optical design utilizing a PC group to expand the FOV and reduce aberrations in compact imaging systems [20]. Our approach differs from recent meta-optical eyepiece designs and advanced freeform surface systems by utilizing conventional optical elements in a novel configuration that maintains manufacturability while achieving superior performance. The novelty of our design lies in the incorporation of an additional PC group, which enhances the FOV while significantly reducing common optical aberrations. Unlike conventional systems that struggle with distortion and field curvature, the PC group enables a unified optimization of the objective and eyepiece groups, resulting in improved image quality across the entire field of view. Furthermore, it facilitates a compact design without compromising performance, making it particularly advantageous for applications requiring thorough scanning of the observation area, where the observation device is permanently fixed and stationary (the device does not move during the scan) to the observation area. The observer scans from one side to the other of the observation area using only eye movement, ensuring that the center of the observer’s scanning line of sight is positioned to maximize both spatial and spectral resolution. Our approach allows for high spatial and spectral resolution across the entire wide FOV. The PC group, which is incorporated into the system, effectively corrects aberrations, resulting in a significantly larger FOV compared to traditional designs with similar specifications which are limited in their FOV due to inherent aberrations in the eyepiece lens. The PC group, which may have positive, negative, or neutral optical power, is strategically placed between the objective and the intermediate image. It acts as an aberration corrector for the eyepiece group, enabling a wider FOV without compromising image quality. In some sense, the PC group may be regarded as a more sophisticated Smyth-lens that does more than just reduce the field curvature and enhance the ER. In a different sense, the PC group may be regarded as a part of a rather complex objective design.

## 2. Materials and Methods

### 2.1. Power-Corrector Group Design Methodology

**Figure 3:**
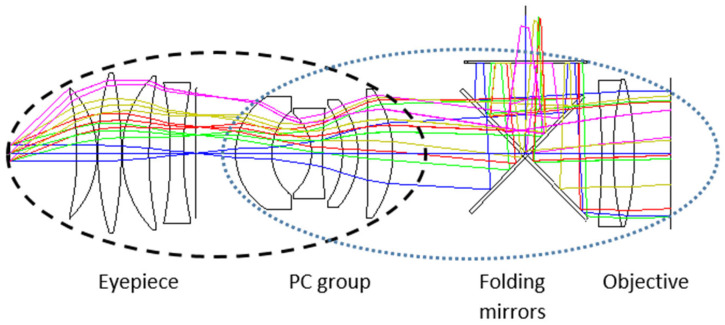
Top view layout of a folded design of a telescope with the PC group. The colored rays represent ray tracing for different field angles.

The PC group is an additional set of lenses positioned between the objective and the intermediate image, as illustrated in Figure 3, which shows a typical design of a folded telescope incorporating a PC group. By integrating the PC group into the optical design, the functionality of the eyepiece and objective groups is unified into a coherent system, providing greater flexibility for design optimization. This unified approach aligns with contemporary optical design trends for global system optimization rather than component-level optimization [6]. Such flexibility enhances control over the entrance and exit pupil sizes, which is essential for achieving a larger FOV without increasing the diameter of the eyepiece elements.

The ellipses in the figure highlight the critical optical interfaces among the three main groups: where the eyepiece group combines with the PC group and where the PC group interfaces with the objective group. The PC group shares ellipses with both the objective and eyepiece groups, reflecting its dual role in the system: it works with the objective to jointly define the system’s input focal length, while also functioning with the eyepiece to correct optical aberrations. This dual functionality is reflected in its name “Power Corrector”—“Power” referring to its combined focal power with the objective, and “Corrector” indicating its primary role in aberration correction with the eyepiece. This illustration demonstrates how these three optical subsystems work together to achieve superior aberration correction and an expanded FOV while maintaining compact dimensions in the overall optical design.

### 2.2. Design Methodology

The main idea of our approach is to predefine all the optical parameters of the system and perform global optimization.

The design process follows the standard procedure for visual observation devices: before starting the design, all optical and mechanical requirements must be gathered, defined, and understood, including magnification, field of view, f-number, diameter, length, weight, and depth of field. Additionally, the maximum constraints for various parameters must be established, such as spectral and spatial resolution at the center of the field, at 0.7 of the field, and at the edge of the field, as well as maximum field curvature, maximum distortion, maximum vignetting, and maximum transmittance. The PC group, located in the middle of the optical device, significantly aids in meeting these design requirements. The global design is carried out in several iterations. In the first stage, an educated guess is made regarding the number of lenses in each group: objective, PC, and eyepiece. The focal lengths of each group are determined, and together with the inversion unit, the diameter of the elements and the overall length are constrained. The spectral range is within the visible spectrum, corresponding to the spectral response of the human eye. A first global optimization is performed.

Based on the results of the first global optimization, adjustments are made for the second global optimization: the focal lengths and the number of lenses in each group are refined, and the maximum design constraints are roughly established. A second global optimization is performed.

Following the results of the second global optimization, adjustments are made for the third global optimization: the focal lengths and the number of lenses in each group are finalized, and the maximum design constraints are precisely established. A third global optimization is performed, and if necessary, additional global optimization may be conducted.

Notably, the PC group is not treated as a special group in global optimization, nor can a specific lens with a unique function be defined. At first glance, the PC appears to function as a field lens, but in reality, it contributes to the overall design performance far beyond that.

It is worth mentioning that, for simplicity, throughout the article, the lenses are treated as spherical lenses. However, there is no limitation on implementing other types of lenses, such as aspheric lenses, in some cases. While our current design utilizes spherical surfaces for manufacturability, the methodology could be extended to incorporate freeform surfaces or aspheric elements to achieve even better performance.

This approach enhances flexibility and performance by providing more options for determining the entrance pupil, exit pupil, and aperture stop diameter. Consequently, the PC group enables a significant expansion of FOV while effectively minimizing the telescope’s aberrations. This allows for a practical eyepiece diameter, making it feasible to use a pair of such telescopes as compact binoculars. Therefore, telescopes incorporating a PC group can be considerably more compact compared to other optical systems with similar performance levels.

The proposed optical design is tailored for specific configurations of objectives, Power-Correctors, and eyepieces. While this customization limits component interchangeability, it reflects a common industry trend toward optimized performance. Such integration enhances optical quality and aligns with manufacturers’ practices to meet specialized user needs, making it a prevalent approach in modern optical design.

## 3. Results

### 3.1. Design and Performance

To demonstrate the potential of the PC group to achieve a wide FOV while maintaining the performance, we have designed a comparison design—an optimized Keplerian telescope with large FOV that includes a PC group and an optimized telescope without the PC group but with identical parameters: the same magnification, apparent FOV, eyepiece focal length, exit pupil diameter, number of lenses, and total length. In our design, for both telescopes, the apparent FOV was chosen to be 86° and the magnification to be 8×—while all the diameters of the eyepiece group were constrained to be no greater than three times the focal length of the eyepiece group to maintain a compact configuration while achieving the desired FOV.

**Figure 4:**
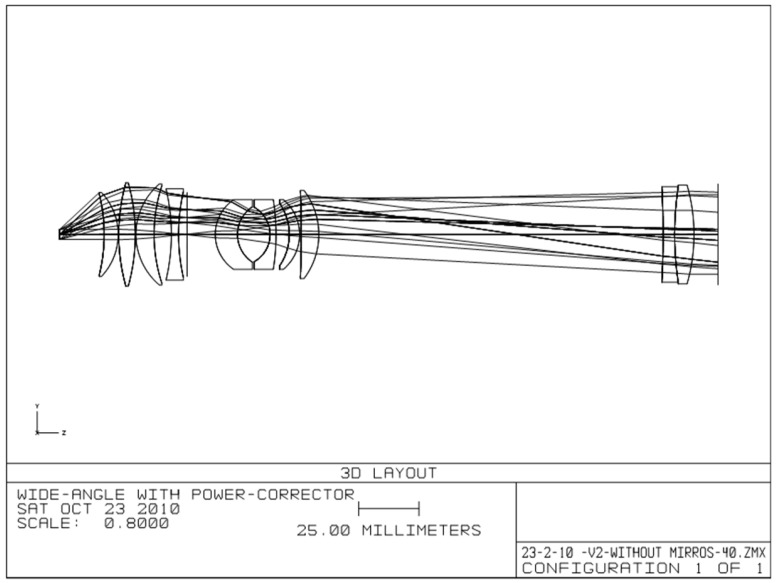
The layout of the telescope with the PC group.

The layout and the surface data summary of the telescope with the PC group are shown in Figure 4 and Table 1, respectively. The maximum diameter of a single lens in the eyepiece reaches 42mm, which is reasonable given that the field of view (FOV) from the eyepiece is 86°, compared to the typical 60–65° in standard binoculars. This design effectively accommodates eye relief (ER) for eyeglass wearers. Notably, there are no specific limitations regarding the f-number, which is an important parameter that should be defined at the beginning of the design process, along with other critical parameters such as magnification and FOV. A specific f-number (F/#) was determined for both designs (with and without the PC) based on the eyepiece configuration, yielding F/# ≈ 4.5, which is within the typical visual-system range of 4–5.

**Figure 5:**
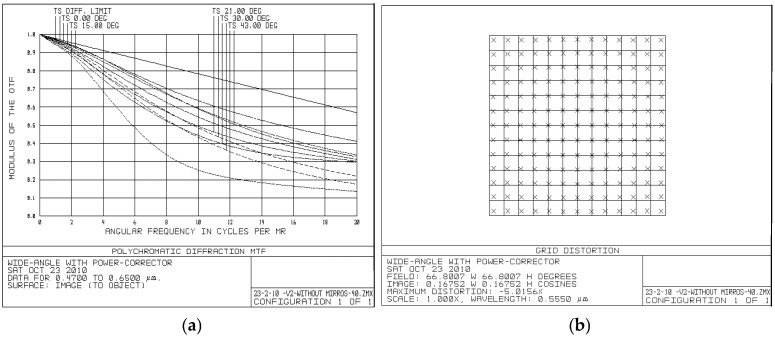
Performance characteristics of the Keplerian telescope design with the PC group: (**a**) the modulation transfer function (MTF); the MTF components are shown across field angles, and (**b**) distortion; distortion is illustrated by crosses, indicating the deviation of chief rays from the grid points that represent the ideal image points.

Figure 5 presents the performance of the telescope with the PC group, where (a) shows the modulation transfer function (MTF); and (b) shows the distortion. The ray tracing is left-to-right from the eyepiece to the objective; therefore, the horizontal axis of the MTF curve represents the angular frequency from the objective to the field. This afocal system preserves parallel beams at varying field angles, so the horizontal axis spans angular frequencies across the field with tangential and sagittal components noted as T and S. The vertical axis in Figure 5a shows the modulus of the OTF. The curves depict the MTF for points at different field angles, with the top line indicating the diffraction limit. The distortion in Figure 5b is shown by crosses, indicating the deviation of chief rays from the grid points that represent the ideal image points.

**Figure 6:**
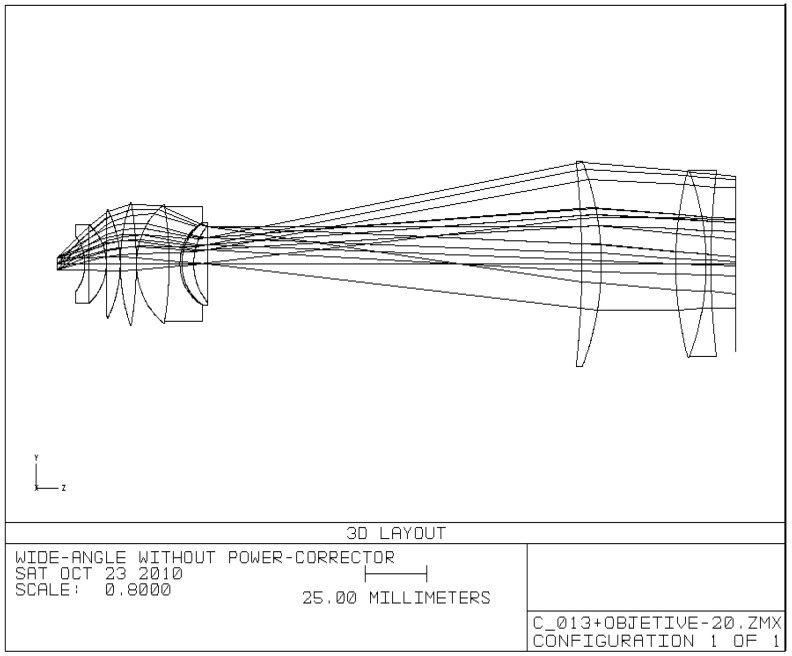
The layout of the telescope without the PC group.

As a comparison, the layout and the surface data summary of the telescope without the PC group are shown in Figure 6 and Table 2, respectively.

**Figure 7:**
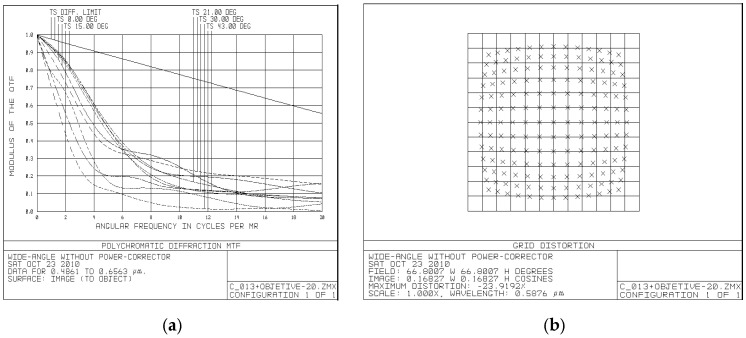
Performance characteristics of the telescope design without the PC group: (**a**) MTF; (**b**) distortion.

Figure 7 presents the performance of the telescope without the PC group, where Figure 7a shows the MTF, and Figure 7b shows the distortion.

Comparison results demonstrate that the telescope with the PC group exhibits superior MTF, significantly reduced distortion level (approximately 5% compared to around 24% without the PC group), and the diameters of the objective lenses are smaller.

### 3.2. Enhanced Compactness and Field of View Performance

To quantitatively evaluate the advantages of using the PC group, we compared a system design that includes the PC group with a system that uses a Nagler-based eyepiece under identical optical parameters: an effective focal length of 19 mm, an exit pupil diameter of 4.2 mm, and a FOV of 86°.

**Figure 8:**
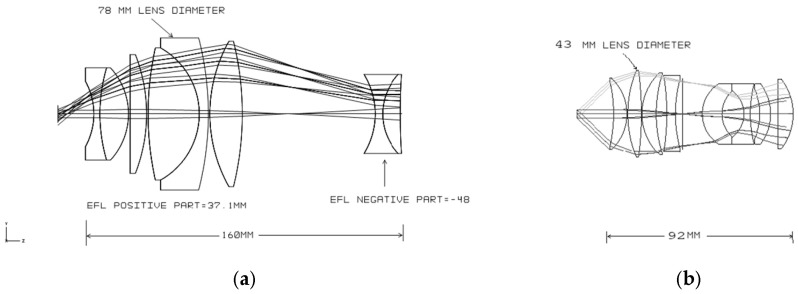
Comparison of optical designs with identical parameters (EFL = 19 mm, Exit Pupil = 4.2 mm, FOV = 86°): (**a**) traditional Nagler-based eyepiece configuration; (**b**) our compact PC-based design.

The comparison is illustrated in Figure 8, where Figure 8a presents the Nagler-based eyepiece and Figure 8b shows our PC-based design.

The dimensional comparison reveals significant size reductions achieved by our PC-based design. While the Nagler-based eyepiece has a total length of 160 mm and maximum diameter of 78 mm, our design achieves the same optical performance with a length of just 92 mm and maximum diameter of 43 mm, representing reductions of 42.5% and 44.9%, respectively. Additionally, our design offers slightly improved eye relief of 15.78 mm compared to 14 mm in the Nagler configuration. Notably, when incorporating the objective lens, which adds approximately the same length to both configurations, the relative size advantage of our PC-based design remains unchanged, maintaining the significant reduction in overall dimensions compared to the Nagler-based system.

### 3.3. Comparison of Field of View Performance Between PC-Equipped and Standard Monoculars

**Figure 9:**
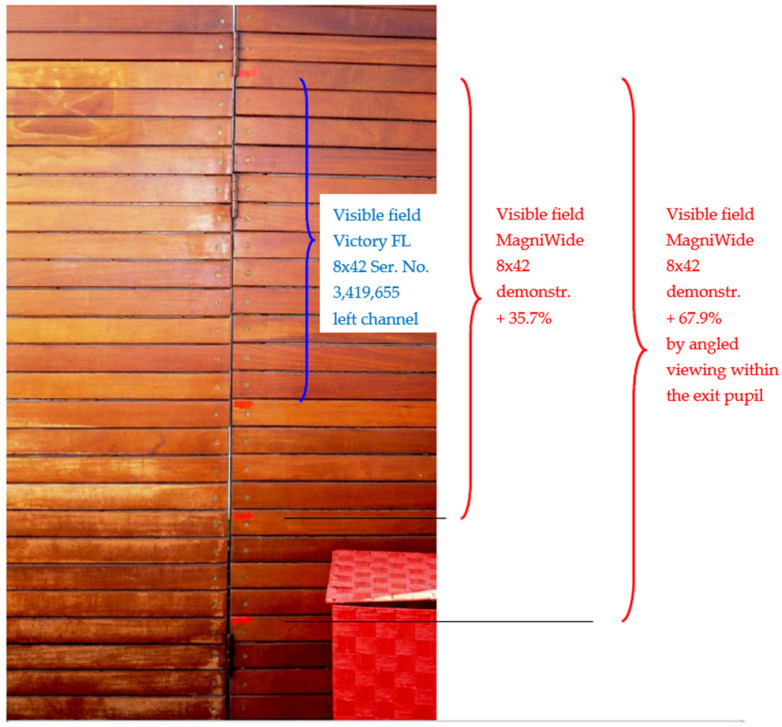
Visible field of view comparison: commercial Zeiss Victory FL 8×42 monocular vs. our PC-equipped 8×42 demonstrator. The demonstrator offers a 35.7% larger field at the same magnification; at a viewing angle within the exit pupil, the field increases by 67.9%, corresponding to roughly a 2.8× enlargement of the viewing area.

To demonstrate the advantage of enlarging the FOV, we compared a monocular design equipped with a PC group to a commercial Zeiss Victory FL 8×42 monocular manufactured by ZEISS, Jena, Germany, both having identical physical dimensions and magnification. Figure 9 compares the visible field of view between the Zeiss Victory FL 8×42 monocular and our demonstrator 8×42 monocular. The demonstrator provides a larger visible field, exceeding Zeiss by 35.7% under equivalent magnification. When the observer’s eye is moved within the exit pupil to view at a viewing angle, the demonstrator’s visible field increases by 67.9%, illustrating the enhanced field performance achievable with the integrated PC group while preserving a compact form factor. This corresponds to enlarging the viewing area by approximately 2.8 times.

While this article provides a foundational understanding of the PC group technology, it is important to note that performance and analyses are highly dependent on the specific design requirements established at the outset. Given the virtually infinite possibilities for optical designs, each with unique performance characteristics and constraints, including performance metrics or simulations for varying system configurations could enhance the applicability of our findings to different optical setups.

## 4. Discussion

The results demonstrate that the incorporation of the PC group provides significant advantages in optical system design. A comparison between an optimized Keplerian telescope with large FOV that includes a PC group and an optimized telescope without the PC group but with identical parameters shows superior MTF performance and dramatically reduced distortion levels (5% versus 24%) indicate that the PC group effectively addresses the fundamental limitations of conventional eyepiece designs. These performance improvements offer practical manufacturing advantages over more complex approaches such as meta-optics or advanced freeform surfaces. This improvement stems from the global optimization approach where the PC group works in conjunction with both the objective and eyepiece to create a unified optical system.

A comparison between a system design that incorporates the PC group and a Nagler-based eyepiece under identical optical parameters shows notable size-reduction achievements. Specifically, the PC-group system achieves a 42.5% reduction in length and a 44.9% reduction in diameter compared to Nagler-based systems, representing substantial improvements in overall compactness. This is particularly significant for portable optical instruments where weight and volume are critical factors.

Comparing a demonstrator equipped with a PC group to a commercial Zeiss Victory FL 8×42 binocular (measured by left monocular only), both having identical physical dimensions and magnification, the demonstrator achieves a 1.679× larger FOV diameter, corresponding to ~2.8× larger viewing area, addressing the fundamental trade-off between magnification and field of view. This improvement has direct practical implications for applications requiring wide area surveillance or observation.

However, the PC group approach also introduces certain limitations. The integration of additional lens elements may result in increased weight, reduced light transmission, and potential contrast degradation due to additional optical interfaces. With appropriate anti-reflective coatings, these losses can be mitigated without compromising the aberration-correction benefits.

More significantly, the system’s requirement for integrated optimization means that standard eyepieces cannot be interchanged with PC-optimized designs, limiting system modularity. To broaden applicability, we propose: (i) a standardized mechanical/optical interface for PC–eyepiece assemblies, (ii) a family of adaptive PC groups tuned for a small set of widely used eyepieces, and (iii) a joint optimization framework that can evaluate alternative eyepieces within a constrained design space.

Future research should focus on developing lightweight materials and advanced optical coatings to mitigate the potential disadvantages while exploring ways to maintain some level of component interchangeability without compromising the system’s integrated optimization benefits.

## 5. Conclusions

This paper presents an advantageous approach to optical design for visual imaging systems through the incorporation of an additional PC group. This design significantly enhances performance and form factor, achieving a FOV diameter that is 1.679 times larger than that of conventional designs with the same physical dimensions and magnification, translating to an approximately 2.8 times larger viewing area.

In detailed comparisons, our PC-based system demonstrated superior modulation transfer function (MTF) and significantly reduced distortion levels (5% versus 24%) for a wide FOV when compared to a conventional design with identical optical parameters. Additionally, greater compactness is achieved compared to Nagler-based systems with the same effective focal length (EFL = 19 mm), exit pupil diameter (4.2 mm), and FOV (86°). Specifically, our design achieved a remarkable reduction in length (42.5%) and diameter (44.9%) while maintaining equivalent optical performance.

The integration of the PC group not only allows for a larger field of view but also maintains image quality by effectively correcting aberrations that typically limit traditional eyepiece designs. However, the inclusion of additional lens elements may lead to increased weight, lower light transmission, and reduced contrast due to reflections and absorption. While these potential drawbacks exist, the incorporation of the PC group substantially enhances optical performance and expands the field of view, underscoring its value in modern optical systems. Moreover, the incompatibility with standard eyepieces is a significant limitation; the PC lens and eyepiece must be corrected and optimized together as an integrated system for optimal performance. As a result, standard eyepieces may not be suitable for use with this specialized PC lens design. To fully leverage the advantages of the PC group, further research is necessary to explore lightweight materials, improve optical coatings, and enhance compatibility with existing eyepiece designs. Furthermore, the incompatibility with standard eyepieces presents a significant limitation; the PC lens and the eyepiece must be corrected and optimized together as an integrated system to achieve optimal performance. Consequently, standard eyepieces are likely not compatible with this specialized PC lens design.

The demonstrated advantages of the PC group technology suggest promising applications in telescopes based on products such as monoculars, binoculars (for bird watching, hunting, sport, military, and astronomical use), and spotting scopes. Future developments may benefit from integration with emerging technologies such as computational imaging and advanced meta-optics to further enhance system capabilities.

## Figures and Tables

**Table 1:** The surface data summary of the telescope with the PC group.

Surf	Comment	Type	Radius	Thickness	Glass	Diameter
OBJ		STANDARD	Infinity	Infinity		0
STO	Exit Pupil	STANDARD	Infinity	18.5		4.2
2	Eyepiece lens 1	STANDARD	−69.99975	5.807029	N-LASF43	33
3	Eyepiece lens 1	STANDARD	−25.44476	0.2402004		35
4	Eyepiece lens 2	STANDARD	79.79214	6.887695	N-LAK9	43
5	Eyepiece lens 2	STANDARD	−79.79214	0.2499943		43
6	Eyepiece lens 3	STANDARD	27.59204	7.534562	N-SF1	42.09881
7	Eyepiece lens 3	STANDARD	65.36421	7.185632		40.5138
8	Eyepiece lens 4	STANDARD	−81.64127	3	N-BASF2	38.04538
9	Eyepiece lens 4	STANDARD	81.64127	3.5		35.50076
10	intermediate image	STANDARD	Infinity	11.72949		34.88509
11	PC lens 5	STANDARD	17.6026	9.235033	N-SF57	29
12	PC lens 5	STANDARD	13.83732	13.52949		23
13	PC lens 6	STANDARD	−12.99517	2.48984	N-LAF7	22.616
14	PC lens 6	STANDARD	−91.26436	5.492719		29
15	PC lens 7	STANDARD	−23.0725	4.277042	N-SF5	26
16	PC lens 7	STANDARD	−18.97247	0.6944073		29
17	PC lens 8	STANDARD	−457.7323	7.284854	N-BASF64	35.774
18	PC lens 8	STANDARD	−30.42477	143		36.67637
19	Objective lens 9	STANDARD	−381.2483	4.796475	N-SF6	38.49232
20	Objective lens 10	STANDARD	133.6307	8.012712	N-BAK2	39.79242
21	Objective lens 10	STANDARD	−75.35432	10		41.15424
IMA	To object	STANDARD	Infinity			41.97317

**Table 2:** The surface data summary of the telescope without the PC group.

Surf	Comment	Type	Radius	Thickness	Glass	Diameter
OBJ		STANDARD	Infinity	Infinity		0
**STO**	Exit pupil	STANDARD	Infinity	11.22352		4.2
**2**	Eyepiece lens 1	STANDARD	−15.9638	1.471002	SF12	20.16894
**3**	Eyepiece lens 2	STANDARD	343.0037	7.166386	SK16	28.65183
**4**	Eyepiece lens 2	STANDARD	−18.3646	0.1		29.18207
**5**	Eyepiece lens 3	STANDARD	1020.46	5.542679	SK16	39.27781
**6**	Eyepiece lens 3	STANDARD	−42.42625	0.1		39.78866
**7**	Eyepiece lens 4	STANDARD	65.24167	6.441244	SK16	44.99595
**8**	Eyepiece lens 6	STANDARD	−125.3955	0.08242904		45.03692
**9**	Eyepiece lens 5	STANDARD	27.17794	13.26808	SK16	43.59262
**10**	Eyepiece lens 6	STANDARD	−118.7091	4.314647	SF12	42.57508
**11**	Eyepiece lens 6	STANDARD	17.23902	0.8302175		30.22803
**12**	Eyepiece lens 7	STANDARD	17.74849	4.768924	SF12	30.44236
**13**	Eyepiece lens 7	STANDARD	20.49193	5.827407		28.59791
**14**	Intermediate image	STANDARD	Infinity	151.5005		28.59794
**15**	Objective lens 8	STANDARD	−323.258	7.926562	N-LASF31A	74.83004
**16**	Objective lens 8	STANDARD	−96.83467	30.21642		75.60162
**17**	Objective lens 9	STANDARD	123.8577	12.32034	N-K5	68.80706
**18**	Objective lens 10	STANDARD	−89.16053	1.912899	P-SF8	68.52388
**19**	Objective lens 10	STANDARD	252.4839	10		66.56563
**IMA**	to object	STANDARD	Infinity			64.54548

## Data Availability

The data that support the findings of this study are included within the article. No additional data were generated or analyzed during the current study.

## Abbreviations

The following abbreviations are used in this manuscript:

**Abbreviation**	**Definition**
PC	Power-Corrector
FOV	Field of View
ExP	Exit Pupil
ER	Eye Relief
MTF	Modulation Transfer Function
EFL	Effective Focal Length
